# Determinants of graft patency after coronary artery bypass grafting

**DOI:** 10.3389/fcvm.2026.1875999

**Published:** 2026-08-21

**Authors:** Hao-Hui Ti, Sheng-Bo Gao, Yu-Ting Wu, Sheng-Jun Dong

**Affiliations:** Department of Cardiovascular Surgery, Shandong Medical and Pharmaceutical University Hospital, Binzhou, Shandong Province, China

**Keywords:** coronary artery bypass grafting, endothelial dysfunction, graft patency, nitric oxide, oxidative stress, reactive oxygen species, saphenous vein graft

## Abstract

Coronary artery bypass grafting (CABG) remains central for multivessel coronary artery disease, but durable benefit depends on graft patency. Patency reflects interacting anatomical, technical, haemodynamic, biological and patient-level determinants. The endothelium offers a useful, metabolically active interface because it regulates vascular tone, thrombosis, inflammation, leukocyte trafficking and repair. This Review examines how conduit choice, harvesting, preservation, anastomotic geometry, target anatomy, operative strategy and comorbidity drive early thrombosis, intermediate intimal hyperplasia and late atherosclerosis. Particular emphasis is placed on oxidative stress, endothelial nitric oxide synthase dysfunction, reduced endothelial nitric oxide (eNO) bioavailability and pathological reactive oxygen species (ROS) signalling. Accordingly, we focus on endothelial nitric oxide synthase (eNOS) dysfunction, reduced eNO bioavailability and pathological ROS signalling, and present an eNO/ROS-centred research framework, requiring local delivery, temporal control and CABG-specific validation.

## 1 Introduction

**Figure 1:**
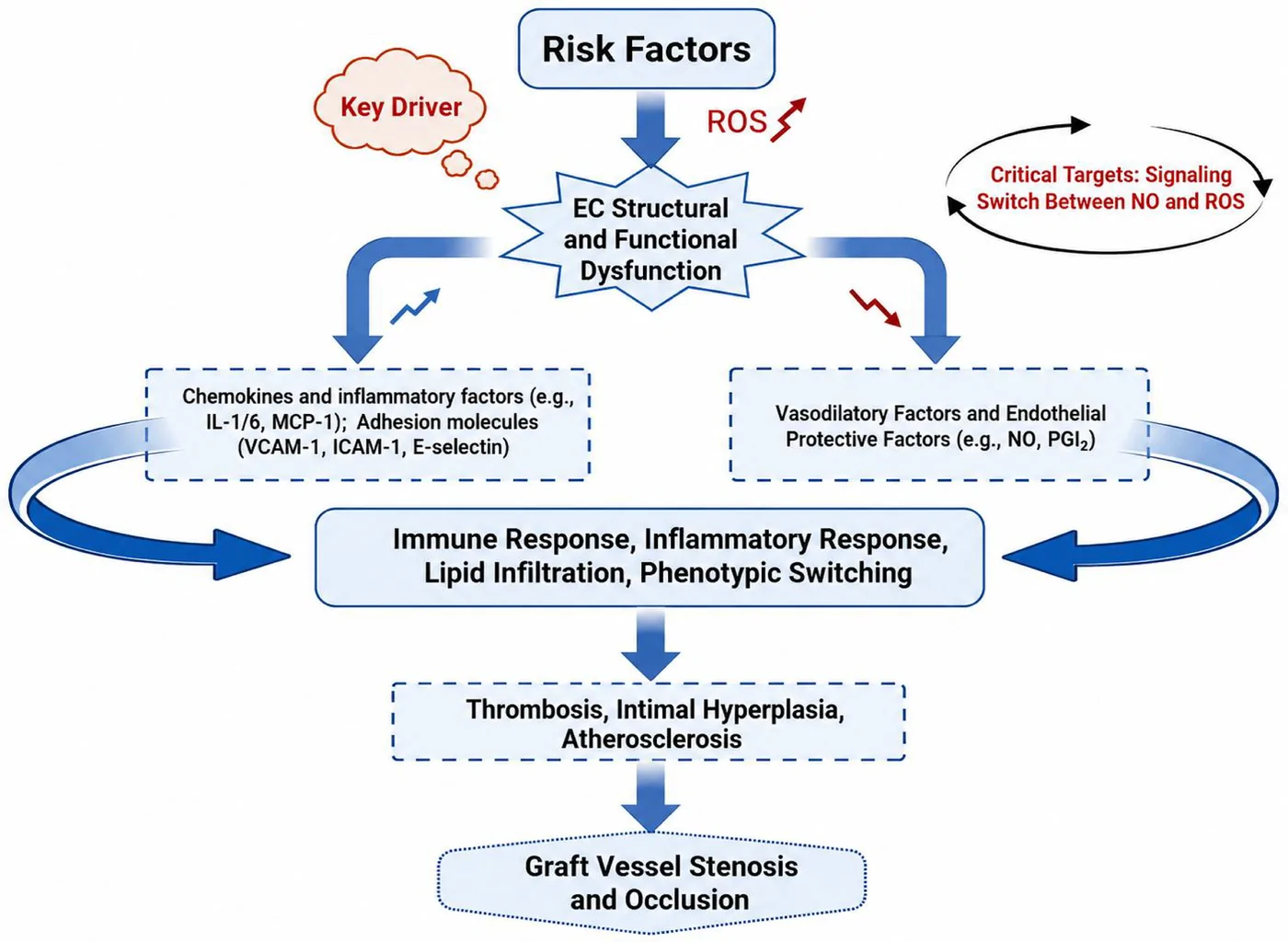
Pathological sequence of graft vasculopathy. Perioperative injury, haemodynamic stress and comorbidity converge on endothelial dysfunction, causing early thrombosis, intermediate intimal hyperplasia and late atherosclerotic remodelling. The redox switch is one mechanism within a multifactorial process.

CABG restores myocardial perfusion by bypassing atherosclerotic coronary stenoses or occlusions with autologous conduits. Although safe and effective, long-term benefit depends on sustained graft patency (1). Failure encapsulates conduit biology, target anatomy, competitive flow, anastomotic precision, preservation injury, thrombosis, inflammation and risk-factor control (Figure 1).

Endothelial injury is a valuable organizing principle because these insults converge at the blood-vessel interface. Implantation exposes grafts to trauma, ischaemia-reperfusion, altered shear, pulsatile pressure, inflammation and metabolic stress. These stimuli reduce eNO bioavailability, amplify ROS and disrupt antithrombotic, vasodilatory and reparative endothelial programmes, including endothelial-to-mesenchymal transition (EndMT) (2, 3). Consequences include platelet deposition, leukocyte adhesion, smooth-muscle-cell migration, intimal hyperplasia, lipid accumulation and atherosclerosis. This Review synthesizes clinical determinants before considering eNO/ROS-targeted interventions as translational opportunities.

## 2 Conduit selection and graft preparation

### 2.1 Conduit selection

Common CABG conduits include the internal thoracic artery (ITA), saphenous vein (SV), radial artery (RA) and right gastroepiploic artery (RGEA). Conduit choice strongly shapes durability. The comparative features of the major CABG conduits are summarized in Table 1. Arterial grafts tolerate systemic pressure and pulsatile shear better than venous grafts because of their wall architecture, vasomotor capacity and endothelial phenotype. By contrast, saphenous vein grafts (SVGs) are abruptly arterialized, predisposing them to over-distension, altered shear, endothelial damage, thrombosis and intimal hyperplasia.

**Table 1:** Comparative profiles of major CABG conduits.

Conduit	Harvest	Patency	Advantages	Limitations	Status
ITA	Pedicled or skeletonized	LITA-LAD generally >90% at 10 years	Best durability; strong endothelial NO; low AS susceptibility	Limited length; BITA may increase sternal risk; harvest method debated	First-line; BITA/MAG individualized
SV	Open, endoscopic or no-touch	Conventional SVG 50–60% at 10 years; NT may improve patency	Long, available, easy to harvest and anastomose; low spasm	Endothelial injury, thrombosis, intimal hyperplasia and AS	Most used; NT and external support under evaluation
RA	Open or endoscopic	Often 80–90% at 10 years	Preferred second arterial conduit; good diameter match; superior to SV	Spasm-prone; avoid with poor hand collateral flow or future dialysis access	Selection and antispasm protocols need optimization

The left internal thoracic artery (LITA) remains preferred, with 10-year patency exceeding 90% in many series. Patency is generally lower for RA, right internal thoracic artery (RITA), RGEA and SV grafts, although estimates vary with target vessel, stenosis severity, imaging method and follow-up completeness (4–6). The RA is the leading second arterial conduit, especially in diabetes or high-grade target stenosis (6–11). Its smooth-muscle-rich media renders it spasm-prone. Calcium-channel blockers have been associated with improved mid-term RA outcomes, but evidence remains mixed and antispasmodic regimens should be individualized (12, 13).

SVGs remain widely used because they are available, familiar, easily anastomosed and relatively spasm-resistant; improving SVG preservation and adaptation therefore remains central to CABG durability.

The arterial-graft advantage is also biological. ITA endothelium generates robust eNO through eNOS, and favourable ITA histomorphology may limit maladaptive intimal thickening (14). eNO regulates vasomotor tone, inhibits platelet adhesion and aggregation, dampens inflammation, promotes repair and suppresses vascular smooth muscle cell (VSMC) proliferation and migration (15, 16). These effects are weaker in SVGs, favouring spasm, intimal hyperplasia and late atherosclerosis.

### 2.2 Harvesting technique

Harvesting determines whether a conduit enters the circulation with an intact endothelium or a prothrombotic surface. Trauma, adventitial stripping, pressure distension and ischaemic storage denude endothelial cells and injure smooth muscle cells. Matrix and tissue-factor exposure trigger coagulation, fibrin deposition, platelet recruitment and leukocyte adhesion, while loss of endothelial vasodilators and antiproliferative mediators favours spasm and intimal hyperplasia (17).

For SVGs, open, endoscopic and no-touch (NT) harvesting are the main approaches. Endoscopic harvesting reduces wound morbidity, but outcomes depend on operator experience and conduit handling (18). NT harvesting preserves perivascular tissue and avoids high-pressure distension, maintaining endothelial integrity and perivascular adipose signalling (19, 20). Evidence is mixed: PATENCY showed lower 3-year vein-graft occlusion with open NT harvest (21, 22), whereas SWEDEGRAFT found no superiority for graft failure or clinical events and more leg wound complications (23). Adoption should reflect wound risk, patient selection, expertise and local practice.

ITA harvesting can be pedicled or skeletonized. Skeletonization increases length and may reduce sternal devascularization, but patency effects remain unsettled. A COMPASS *post hoc* analysis reported higher 1-year ITA stenosis after skeletonization without fully controlling surgeon-level factors (24). Conversely, skeletonization may reduce sternal wound complications in selected patients. Neither approach is universally superior; outcomes depend on patient risk, trauma, handling and operator experience.

RA harvesting may be open or endoscopic. Meta-analytic data suggest endoscopic harvest reduces site morbidity without a clear survival or patency penalty, although smaller physiological studies question endothelial preservation (25). Future comparisons should emphasize endothelial integrity, vasoreactivity and long-term patency, not wound outcomes alone.

### 2.3 External support of saphenous vein grafts

SVG intimal hyperplasia adapts to arterial pressure and shear. External supports aim to limit over-distension, normalize wall stress and restrain intimal growth. Early studies reported less intimal hyperplasia and lumen irregularity, yet a recent North American multicentre trial found no 12-month reduction in intimal-hyperplasia area (26). Differences in device design, territory, implantation, imaging endpoints and follow-up likely explain conflicting evidence. External support is promising but not universally validated.

Perivascular drug delivery may strengthen mechanical support. Intraluminal drug-eluting devices can injure grafts and perform poorly in diffuse disease, whereas adventitial delivery targets the wall more directly (27). Rapamycin-coated biodegradable supports suppress intimal hyperplasia experimentally (28), but clinical SVG benefit remains unproven.

## 3 Preservation solutions and endothelial protection

Ischaemia during preparation and reperfusion after implantation increase ROS and impair endothelial barrier function. Preservation solutions are intraoperative endothelial-protection tools: they should maintain ionic and osmotic stability, prevent spasm, preserve eNO signalling and limit oxidative injury. The 2024 graft-treatment consensus emphasized standardized handling and preparation, highlighting the gap between preservation concepts and routine practice (29). The preservation strategies and their current evidence are summarized in Table 2.

**Table 2:** Preservation solution logic. Preservation strategies differ in buffering, antioxidant and vasodilator capacity. Current evidence supports careful handling and physiological storage, but head-to-head clinical data remain heterogeneous and should be linked to standardized endothelial and angiographic endpoints.

Category	Key features	Research status
Heparinized saline	Simple baseline; heparin reduces thrombosis risk.	Widely used; limited endothelial protection.
AWB (autologous whole blood)	Heparinized AWB at 4 °C may reduce EC injury and oxidative stress.	Common in the UK; spasm and endothelial injury remain concerns.
UWS(University of Wisconsin solution)	Intracellular-type solution with antioxidants and iron chelation.	May protect ECs; high potassium can promote vasoconstriction.
Heparin-papaverine	Papaverine relieves spasm; acidic pH may damage ECs.	Common but suboptimal for endothelial protection.
Tiprotec	Buffered potassium-rich solution with iron chelation and antioxidant support.	Promising preclinical data; limited clinical adoption.
VG (verapamil–nitroglycerin–Ringer)	Verapamil-nitroglycerin-Ringer solution relieves spasm and supports NO signalling.	Increasingly used for RA preservation; needs validation.

### 3.1 Choice of preservation solution

Traditional media, including heparinized saline, blood-based solutions, buffered crystalloids and specialized preparations, differ in pH, osmolarity, antioxidant capacity and vasodilator content. Evidence remains heterogeneous, and few studies link *ex vivo* endothelial markers to long-term angiographic or clinical patency (29–33), so future trials should combine standardized endothelial assays, graft imaging and clinical outcomes.

### 3.2 Endothelial-protective preservation additives

Endothelial-protective additives aim to protect grafts during *ex vivo* storage. DuraGraft®, derived from the GALA formulation, contains reduced glutathione, L-ascorbic acid and L-arginine in Hanks' balanced salt solution, coupling antioxidant protection with substrate support for eNO generation (34–36). Use has expanded internationally, but larger long-term studies must define effects on clinical events and graft patency, especially versus conventional solutions.

### 3.3 Emerging preservation strategies

Heparinized saline with papaverine remains common. Papaverine limits vasospasm, but acidic formulations may damage endothelial ultrastructure (37). Ischaemia-reperfusion also suppresses endogenous nitric oxide (NO) production (37). Verapamil-nitroglycerin (VG) solution combines calcium-channel blockade with an exogenous NO donor, and experimental studies support endothelial preservation and less arterial-graft spasm (33). Overall, buffering, selective antioxidant activity and controlled vasodilation should match the perioperative injury window.

## 4 Surgical strategy and haemodynamic design

### 4.1 Single versus sequential grafting

Sequential grafting conserves conduit length, reduces proximal anastomoses and may shorten operative time. For SVGs, some 10-year data show acceptable safety and better patency than individual grafting (38), but interpretation is limited by selection bias, target quality, run-off, competitive flow and surgeon preference. For LITA, angiographic data suggest single grafts have the best patency, followed by Y-grafts; sequential configurations may risk inflow compromise (39). Design should reflect target territory, vessel size, run-off and flow competition.

### 4.2 Composite grafts and multi-arterial grafting

Arteriovenous composite grafting remains debated. LITA-SV composite grafts may expose the vein to ITA-derived protective mediators and a lower, more physiological pressure profile than an aorta-based SVG. However, competitive flow or steal can impair early patency, especially when the SV is oversized or target stenosis is moderate (40). Diameter matching is important; the SV should ideally not exceed the LITA diameter by more than 50%.

Y- or T-composite SVGs attached to an *in situ* ITA also avoid the high-pressure, turbulent aortic pulse. ITA-regulated flow may reduce acute endothelial denudation and eNOS uncoupling, and imaging/computational studies suggest adaptive SVG remodelling toward arterial dimensions (41, 42). Translation requires caution because performance depends on inflow reserve, stenosis, diameter, run-off and technical precision.

Multi-arterial grafting is increasingly emphasized. In suitable patients, greater arterial-conduit use often outperforms LITA-SVG-only strategies (43). RA-based LITA-RA configurations may offer strong flow reserve and revascularization rates (44, 45). However, the 10-year Arterial Revascularization Trial found no intention-to-treat mortality advantage of bilateral internal thoracic artery (BITA) over single ITA grafting and more sternal wound complications with BITA (46). Multi-arterial grafting should be individualized rather than treated as a default.

### 4.3 Anastomotic quality

Intimal hyperplasia often begins near the anastomosis. Local geometry controls shear stress, turbulence, residence time and platelet activation, making anastomotic design a direct determinant of flow and durability. End-to-side anastomosis can create unfavourable angles, especially when SVG and coronary diameters mismatch. Side-to-side anastomosis may improve flow alignment and address mismatch (47, 48); in sequential grafting, proximal side-to-side sites often remain patent because flow and pressure are higher. Disadvantages are equally important: poor angle or position can create steal, and disease in one conduit segment can threaten downstream territories.

Technical precision remains decisive. Adventitial or fat incorporation, purse-string narrowing, excessive tightness and poor target selection can reduce flow and cause early failure. Intraoperative flow assessment should be a routine quality control measure.

### 4.4 On-pump versus off-pump CABG

Comparative studies report heterogeneous graft-patency findings after on-pump versus off-pump CABG (49). Cardiopulmonary bypass is not the only determinant: minimally invasive extracorporeal circulation may reduce inflammation in selected patients (50), and experienced off-pump programmes can achieve excellent long-term patency (51). The key question is whether the strategy enables complete revascularization, precise anastomoses and reproducible flow. Study design, surgeon experience, conversion rate and completeness must be considered.

## 5 Target-vessel determinants

### 5.1 Degree of stenosis

Target-vessel stenosis influences patency through competitive flow. Severe left anterior descending artery stenosis improves long-term LITA patency by reducing native antegrade flow and increasing graft flow (52). Conversely, grafting moderately stenosed vessels can instead produce low flow, stasis and thrombosis. Target selection should integrate anatomical stenosis, functional severity, myocardial territory and expected run-off.

### 5.2 Target-vessel diameter

Target-vessel diameter is also critical. A calibre above 1.5 mm is generally favourable; in one off-pump series, patency was higher above than below this threshold (96.6% versus 91.1%) (53). Small vessels increase technical difficulty, reduce flow and magnify minor anastomotic imperfections.

## 6 Comorbid disease and postoperative management

### 6.1 Hypertension, hyperglycaemia, hyperlipidaemia and vascular ageing

Hyperglycaemia, dyslipidaemia and hypertension promote graft vasculopathy by converging on endothelial dysfunction, inflammation and oxidative stress.

**Figure 2:**
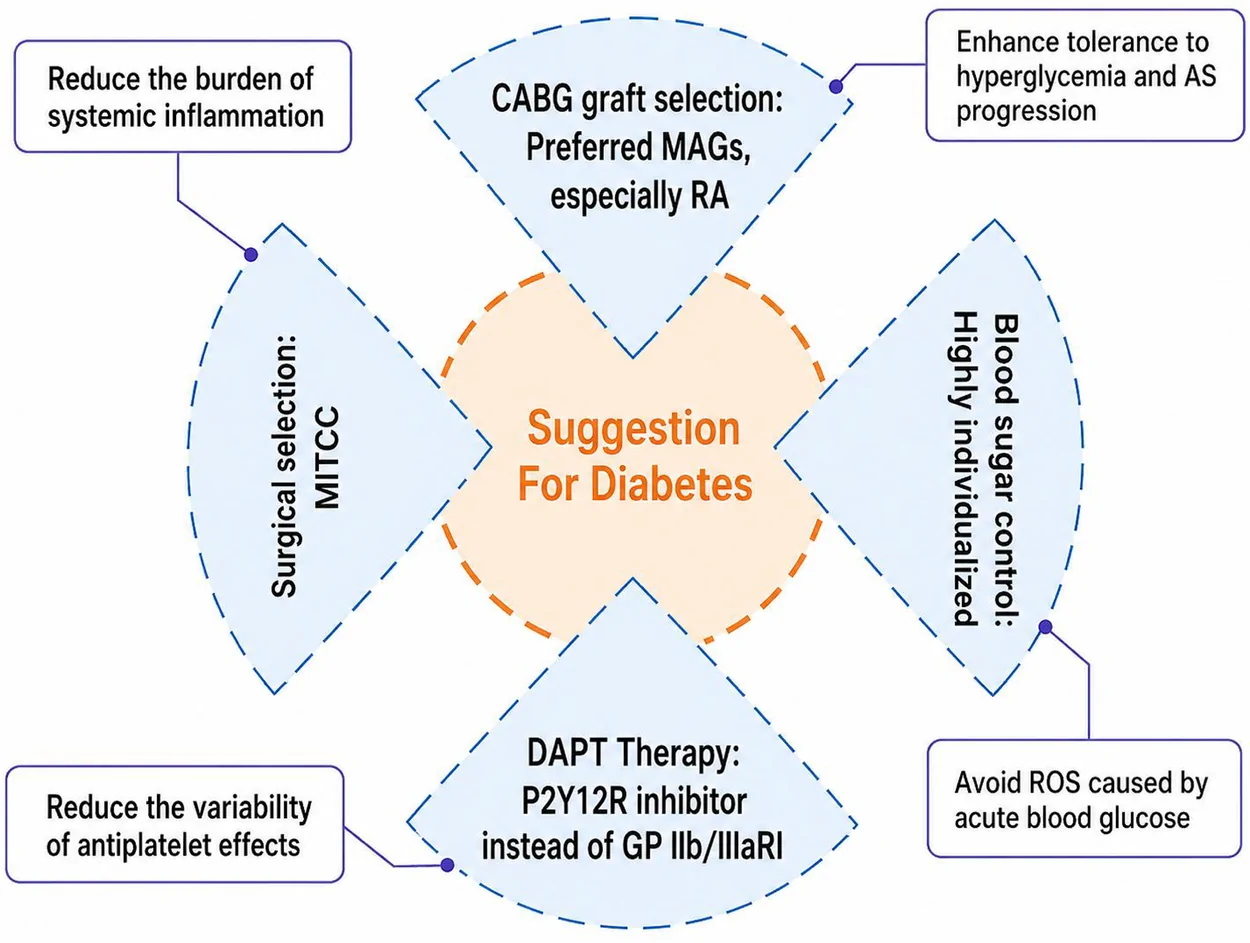
Conduit selection (including multiple arterial grafting/radial artery), individualized glycaemic control, antiplatelet strategy, minimally invasive surgery, and their intended clinical goals.

Diabetes activates the polyol pathway, promotes advanced glycation end product (AGE) accumulation, insulin resistance and metabolic inflammation, and drives eNOS uncoupling. Uncoupled eNOS generates superoxide rather than eNO; superoxide reacts with eNO to form peroxynitrite, further oxidizing tetrahydrobiopterin (BH4) and sustaining endothelial injury (54, 55). Glycaemic control after CABG should be individualized because hypoglycaemia and acute glucose variability can also increase oxidative stress (56) (Figure 2).

Hyperlipidaemia increases viscosity, platelet reactivity, lipid deposition and plaque formation in the graft wall. The Post-CABG trial showed that aggressive low-density lipoprotein cholesterol (LDL-C) lowering delayed SVG atherosclerosis versus moderate lowering (57). Early active lipid-lowering with statins, and when needed ezetimibe or other agents, remains central to secondary prevention (17, 58). However, NEWTON-CABG CardioLink-5 found that evolocumab did not reduce SVG disease at 24 months despite marked LDL-C reduction (59). Lipid control is essential but insufficient for early failure driven by thrombosis, injury and remodelling.

Chronic hypertension increases wall tension, impairs endothelial function and accelerates remodelling through vasoconstrictor-vasodilator imbalance, inflammation and ROS. Ageing adds AGE-mediated collagen cross-linking, stiffness and reduced compliance (60, 61). Graft-native compliance mismatch disturbs flow and promotes focal intimal hyperplasia; reducing mismatch can attenuate anastomotic hyperplasia experimentally (62, 63).

### 6.2 Hypercoagulability and antiplatelet therapy

Hypercoagulability drives early graft thrombosis. Platelet hyperreactivity and elevated coagulation factors increase viscosity and clot formation. Activated platelets release interleukin-1 (IL-1), interleukin-6 (IL-6) and transforming growth factor-*β*, while thrombin and fibrin amplify VSMC proliferation and intimal hyperplasia.

Aspirin remains foundational and should generally continue indefinitely unless contraindicated (58). Dual antiplatelet therapy (DAPT), adding a P2Y12 inhibitor such as clopidogrel or ticagrelor, can reduce SVG failure in selected patients but increases bleeding (64). TOP-CABG showed that 3 months of ticagrelor-based DAPT was non-inferior to 12 months for SVG occlusion and reduced clinically relevant bleeding (65). Therapy should be individualized, particularly in diabetes or acute coronary syndrome, where platelet reactivity and variable clopidogrel response are common (66).

### 6.3 Inflammatory disease

Chronic low-grade inflammatory states, particularly periodontitis, may contribute to systemic endothelial dysfunction through bacteraemia, cytokine release and oxidative stress, but direct evidence linking them to CABG graft patency remains limited (67–69). CANTOS provides proof-of-concept that IL-1β inhibition with canakinumab can reduce recurrent cardiovascular events without lowering LDL-C; however, this evidence derives from non-CABG atherosclerotic populations, and safety and efficacy for CABG graft protection remain unresolved (70, 71).

### 6.4 Gut microbiota dysbiosis

The gut microbiota influences immune homeostasis, metabolism and thrombosis. Dysbiosis and trimethylamine N-oxide (TMAO) are linked to oxidative stress, platelet hyperreactivity and plaque instability (72, 73). TMAO may promote inflammation, endothelial apoptosis and coagulation-factor accumulation. Direct evidence for microbiota-derived metabolites as graft-patency determinants remains limited, so this axis should remain exploratory (74).

### 6.5 Renal insufficiency

Chronic kidney disease accelerates atherosclerosis and worsens outcomes after CABG. Reduced glomerular filtration rate reflects systemic vascular injury, inflammation and intimal hyperplasia (75). Proteinuria may align more closely with endothelial dysfunction than filtration rate in some patients with type 2 diabetes. Albuminuria, rather than estimated filtration rate, has correlated with endothelial function in diabetic nephropathy (76). Low-grade inflammation and insulin resistance likely connect renal injury, proteinuria and vascular dysfunction.

**Figure 3:**
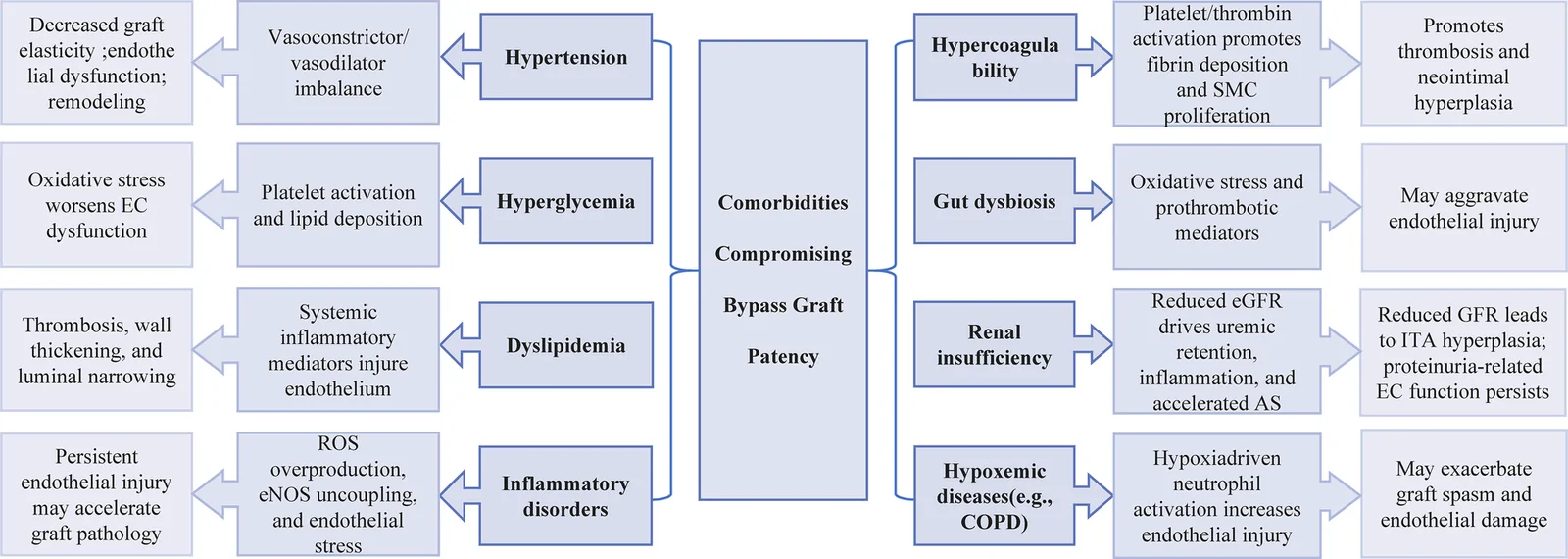
Comorbid disease pathways affecting graft patency. Metabolic, inflammatory, renal, microbiota-related and ageing processes interact through endothelial activation, thrombosis, intimal hyperplasia and atherosclerotic remodelling.

Overall, metabolic disease, inflammation, dysbiosis and renal dysfunction converge on endothelial injury while also acting through AGE formation, lipid deposition, wall stress and platelet activation; management must be integrated and patient-specific (Figure 3).

## 7 Gene-based strategies for graft optimization

Single-cell sequencing has identified conduit-specific programmes relevant to graft optimization. In the RA, macrophage migration inhibitory factor (MIF)-associated signalling may contribute to intimal thickening. In the RGEA, altered calcium-channel gene expression, including CACNA1C, may help explain spasm and relative calcium-channel-blocker insensitivity. CREB5 and GDF10 expression in RA and RGEA may promote extracellular-matrix deposition and fibrosis, whereas lipid-lowering strategies such as proprotein convertase subtilisin/kexin type 9 (PCSK9) inhibition may protect ITA conduits from late atherosclerosis. These associations remain hypothesis-generating until linked to graft-specific functional endpoints (77).

Therapeutic translation remains early. Gene-based interventions face inefficient vascular-wall delivery, limited cell specificity, immune responses, off-target effects, vector toxicity, manufacturing complexity and uncertain long-term safety (78). Adeno-associated viral vectors transduce efficiently but raise immunogenicity and genome-safety concerns (79). Nonviral vectors, including lipid nanoparticles, polymeric carriers and hydrogel-assisted perivascular depots, may be more controllable but still need better targeting, penetration and retention (80).

For CABG, local delivery is most realistic. *Ex vivo* transduction during preparation could expose the conduit to a defined dose before implantation, whereas periadventitial biomaterials could sustain release after anastomosis. Targets should be prioritized only when a validated molecular abnormality maps to measurable graft phenotypes, such as endothelial coverage, vasoreactivity, leukocyte adhesion, VSMC migration or neointimal area. MIF, CACNA1C, CREB5 and GDF10 remain preclinical targets requiring *in vivo* validation, dose-ranging, biodistribution and long-term safety assessment.

## 8 Future perspective: eNO/ROS biology as a translational research framework

### 8.1 Mechanistic rationale

**Figure 4:**
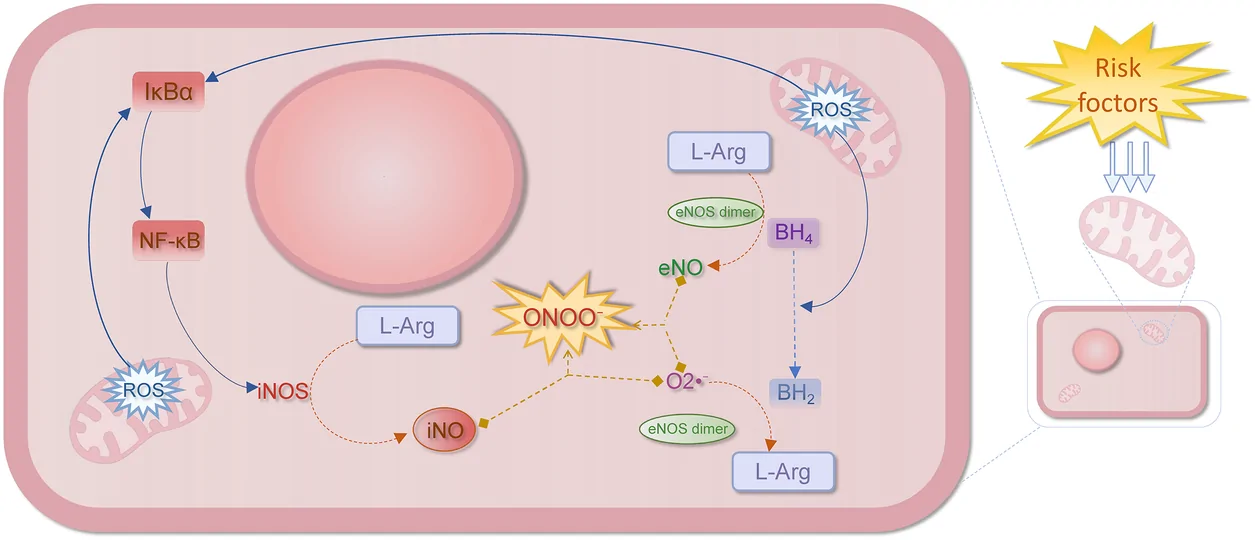
This schematic illustrates nitric oxide (NO) synthesis and dysregulated redox signalling under oxidative stress. After arterial implantation, saphenous vein grafts (SVGs) are exposed to endothelial denudation, platelet-, monocyte- and neutrophil-derived mediators, hypoxia and altered prostacyclin metabolism, which can upregulate or activate NADPH oxidase (NOX). NOX catalyses the single-electron reduction of O₂ to superoxide (O₂•⁻), initiating a pro-oxidant cascade. Under physiological conditions, sufficient L-arginine (L-Arg) and tetrahydrobiopterin (BH₄) allow dimeric endothelial nitric oxide synthase (eNOS) to generate vasodilatory and vasoprotective endothelial NO (eNO). In CABG grafts, however, harvesting trauma, post-harvest ischaemia, reperfusion and arterial haemodynamic stress amplify oxidative stress. Excess reactive oxygen species (ROS) oxidize BH₄ to dihydrobiopterin (BH₂), which fails to stabilize the eNOS dimer. This promotes eNOS uncoupling, disrupts intramolecular electron transfer and diverts electrons from L-Arg to molecular oxygen, generating further O₂•⁻. Thus, eNOS shifts from protective NO production to pro-oxidant superoxide generation, amplifying graft injury. Superoxide dismutase (SOD) dismutates O₂•⁻ to hydrogen peroxide (H₂O₂), which can generate highly reactive hydroxyl radicals (•OH), particularly with redox-active transition metals. These radicals damage proteins, lipids, nucleic acids and mitochondria, while persistent O₂•⁻ sustains ROS amplification and reduces NO bioavailability. In parallel, ROS activate nuclear factor-*κ*B (NF-*κ*B), upregulating inducible nitric oxide synthase (iNOS). Unlike tightly regulated eNOS, iNOS can produce sustained, high-output NO during inflammation. In an O₂•⁻-rich environment, iNOS-derived NO rapidly forms peroxynitrite (ONOO⁻), a potent reactive nitrogen species that further oxidizes BH₄, damages eNOS and aggravates endothelial dysfunction. Together, NOX activation, eNOS uncoupling, ROS amplification and iNOS-dependent nitrosative stress establish a self-reinforcing oxidative-inflammatory cycle that drives SVG dysfunction (81, 82).

The determinants described above do not converge on one dominant mechanism. They interact through haemodynamics, target anatomy, platelets, inflammation, lipid biology, technical precision and endothelial stress. Within this network, oxidative stress-driven endothelial dysfunction is a tractable hub. Excess ROS oxidize BH4, destabilize eNOS dimers and uncouple eNOS; uncoupled eNOS generates superoxide rather than eNO, amplifying endothelial injury, inflammation, intimal hyperplasia and atherosclerosis (81, 82) (Figure 4).

Restoring protective eNO while selectively suppressing pathological ROS may improve endothelial homeostasis more effectively than either approach alone. eNO loss and ROS excess reinforce each other; NO supplementation in a superoxide-rich milieu can increase peroxynitrite formation; and sustained inflammation can convert inducible nitric oxide synthase (iNOS)-derived NO into a cytotoxic signal. Context-dependent iNOS modulation may therefore be needed. This rationale aligns with vascular reviews linking endothelial dysfunction, NO depletion, oxidative stress, inflammation, thrombosis and remodelling (83).

### 8.2 Candidate intervention strategies

Targeted eNO prodrug delivery is attractive but experimental for CABG. Bump-and-hole enzyme-prodrug systems can spatially control NO release in non-CABG models (84), and long-acting donors or microneedle-based biomaterials are being explored (85). For graft protection, the key challenge is local wall targeting without systemic vasodilation, hypotension or excess peroxynitrite formation.

ROS-targeted therapy must be selective. Conventional antioxidants and ROS-responsive nanoscavengers may reduce ischaemia-reperfusion injury, but indiscriminate scavenging could disrupt physiological redox signalling (86). Materials responsive to pathological ROS thresholds, or directed at defined ROS species and sources, may better balance protection with signalling preservation (87, 88). Notably, CABG-specific evidence remains limited.

iNOS modulation may help during inflammatory graft injury. Persistent iNOS activation generates NO that reacts with superoxide to form peroxynitrite, intensifying oxidative and nitrosative damage (89). However, systemic iNOS suppression could impair host defence, so any approach requires local and temporal control.

These approaches are biologically plausible but not evidence-based CABG therapies. Their value lies in combining eNO restoration, selective ROS control and phase-specific inflammatory modulation without abolishing physiological signalling; rigorous validation is required.

### 8.3 Translational challenges

Delivery is the first barrier. Therapeutics must reach injured SVG regions, especially anastomoses and denuded endothelium, at effective concentrations and durations. Systemic delivery is inefficient and off-target. Perivascular nano-engineered biomaterials may improve graft-wall exposure (90), and adventitial nanoparticles have reduced intimal hyperplasia in vascular-injury models (91). Nonetheless, human SVG penetration, retention, biodegradation and clearance remain unresolved.

Safety is equally important. eNO is protective at physiological levels, but in an oxidative milieu excess NO can form peroxynitrite (92). Conversely, complete ROS elimination may impair adaptive signalling and broad iNOS inhibition may increase infection risk. Ideal platforms should respond to local redox or enzymatic cues, titrate release and degrade without chronic toxicity (93).

Furthermore, timing must match biology. Harvest injury and ischaemia-reperfusion occur within hours, whereas SVG arterialization develops over weeks to months. Interventions may require staged intraoperative preservation followed by early postoperative remodelling support.

eNO/ROS-targeted therapy should complement, not replace, established care. It may integrate with preservation solutions, external supports, gene-based approaches and individualized antiplatelet therapy. Hydrogen sulfide (H2S), an endogenous gasotransmitter, may broaden the framework by promoting eNOS phosphorylation, NO production and antioxidant pathways (94, 95). NO-H2S strategies remain experimental and require CABG-specific safety and efficacy testing.

### 8.4 Experimental pathway and validation endpoints

A staged validation pathway is needed. Discarded human SVG segments should first be tested *ex vivo* under standardized storage, distension and inflammatory stimuli, with endpoints including endothelial coverage, acetylcholine-dependent relaxation, NO release, ROS profiling, BH4 oxidation, eNOS dimerization, iNOS induction, platelet adhesion and leukocyte binding. Pulsatile arterial-pressure bioreactors can model arterialization and compare preservation media, periadventitial depots and responsive materials. Animal vein-graft models should evaluate biodistribution, retention, degradation, toxicity, hypotension, immune activation and neointimal area. Early clinical studies should combine mechanistic biomarkers with computed tomography angiography (CTA)-based patency and patient-centred outcomes.

This staged approach separates current evidence from future research. Established practice must remain centred on conduit selection, atraumatic harvesting, standardized preservation, precise anastomosis, graft-flow assessment and secondary prevention; eNO/ROS delivery, iNOS modulation, gene-based strategies and NO-H2S combinations remain experimental.

## 9 Conclusion

Graft patency after CABG reflects interacting conduit, surgical, haemodynamic and patient-level variables. Endothelial dysfunction and oxidative stress are important, but failure cannot be reduced solely to ROS/NO imbalance; competitive flow, target calibre, anastomotic geometry, harvesting injury, preservation, platelet biology, inflammation, lipid control and comorbidity all matter.

The eNO/ROS framework is translational, not therapeutic doctrine. Restoring eNO, limiting pathological ROS and modulating iNOS during inflammatory injury may interrupt oxidative and nitrosative feed-forward loops, but translation requires local delivery, dose-responsive materials, clinically relevant models and trials linking biomarkers to graft imaging and outcomes.

**Figure 5:**
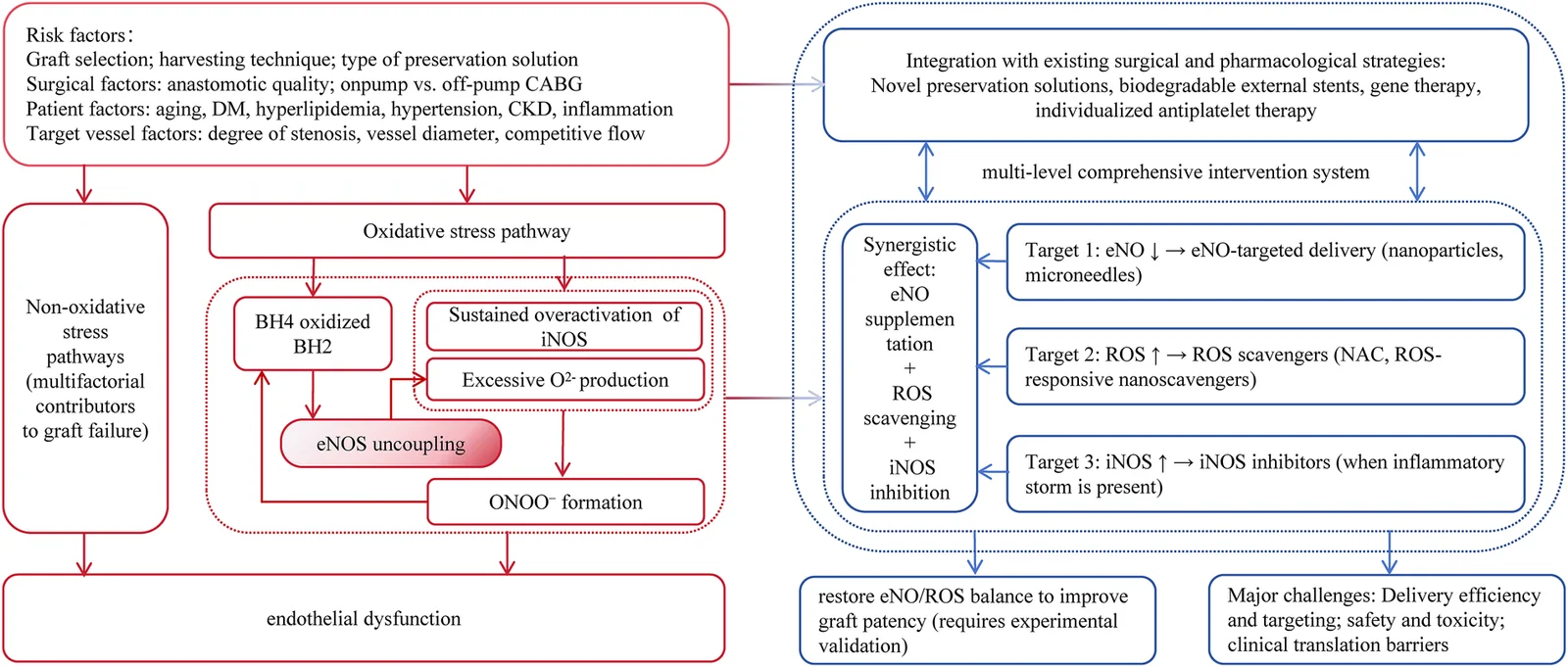
eNO/ROS-centred translational framework for CABG graft protection. Established clinical and technical determinants converge through oxidative stress-dependent and independent pathways to endothelial dysfunction. The framework positions eNO restoration, selective ROS control, contextual iNOS modulation and NO-H2S approaches as experimental strategies requiring CABG-specific validation.

Figure 5 links clinical determinants with endothelial redox biology and positions eNO restoration, ROS control, iNOS modulation and H_2_S-based approaches as future experimental strategies. Immediate priorities remain meticulous conduit handling, rational selection, precise anastomoses, standardized preservation, individualized antiplatelet therapy and sustained secondary prevention.
